# Single-digit clubbing revealing an occult fracture

**DOI:** 10.1093/omcr/omaf065

**Published:** 2025-06-27

**Authors:** Mahesh Mathur, Sushan Adhikari, Sumit Paudel, Nabita Bhattarai, Sambidha Karki, Sandhya Regmi

**Affiliations:** Department of Dermatology, College of Medical Sciences Teaching Hospital, Bharatpur, Nepal; Department of Orthopaedics, Bharatpur Hospital, Bharatpur, Nepal; Department of Dermatology, College of Medical Sciences Teaching Hospital, Bharatpur, Nepal; Department of Dermatology, College of Medical Sciences Teaching Hospital, Bharatpur, Nepal; Department of Dermatology, College of Medical Sciences Teaching Hospital, Bharatpur, Nepal; Department of Dermatology, College of Medical Sciences Teaching Hospital, Bharatpur, Nepal

**Keywords:** clubbing, unidigital clubbing, Hippocratic toes

## Abstract

Single-digit clubbing is a rare physical sign, with only a few cases reported in the literature. The causes of single-digit clubbing are myxoid cyst, osteoid osteoma, enchondromas, myxochondromas, superficial acral fibromyxoma, sarcoidosis, median, and ulnar nerve injury. We hereby report a case of solitary digital clubbing of the toe due to an underlying occult fracture.

## Introduction

Digital clubbing is a clinical sign resulting from a focal bullous enlargement of the terminal segments of the digits due to connective tissue proliferation between the distal phalanx and nail matrix [1]. The clubbing may be symmetric bilaterally, or it may be unilateral or even unidigital [1]. Single-digit clubbing is a rare physical sign, with only a few cases reported in the literature [1, 2]. We hereby report a case of solitary digital clubbing of the toe due to an underlying occult fracture.

## Case presentation

**Figure 1:**
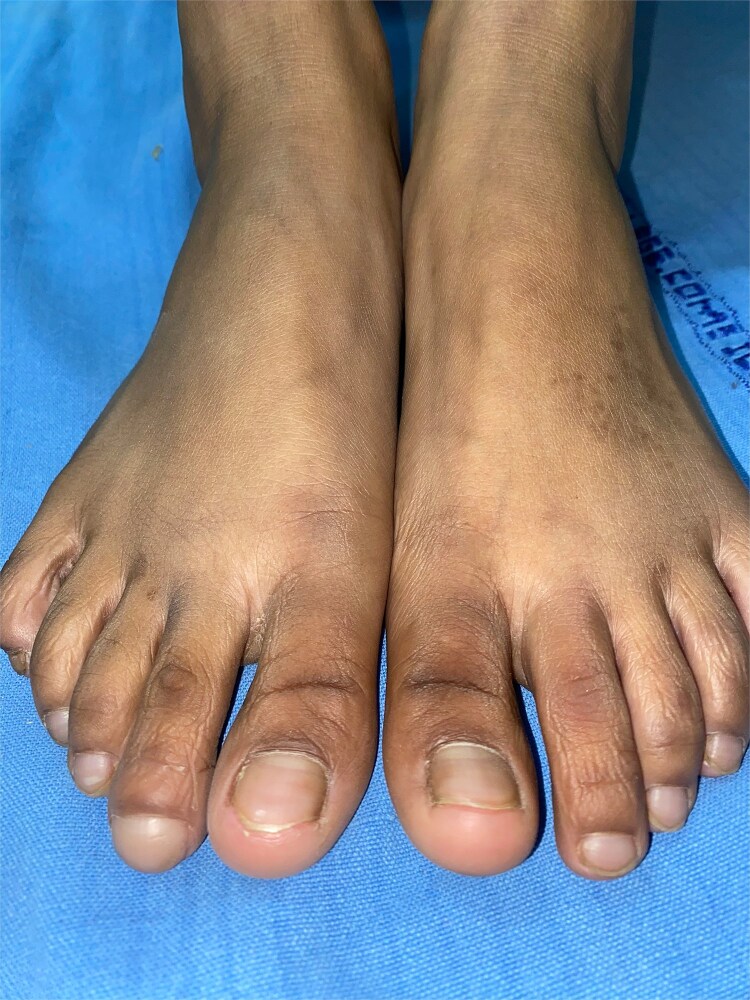
Clubbing in distal phalanx of the second toe of right foot.

A 17-year-old female presented with clubbing in the distal phalanx of the second toe with a Lovibond’s angle greater than 180 degrees for 2 months (Fig. 1). There was gradual enlargement of the toe over time, so she came for dermatology consultation. All other toes were normal. No other family members had similar complaints. She gave a history of local trauma to the right foot 4 months back, but as plain radiographs were normal, she did not receive any treatment. Neurological examination, including sensory and motor examination of the bilateral foot, was normal.

**Figure 2:**
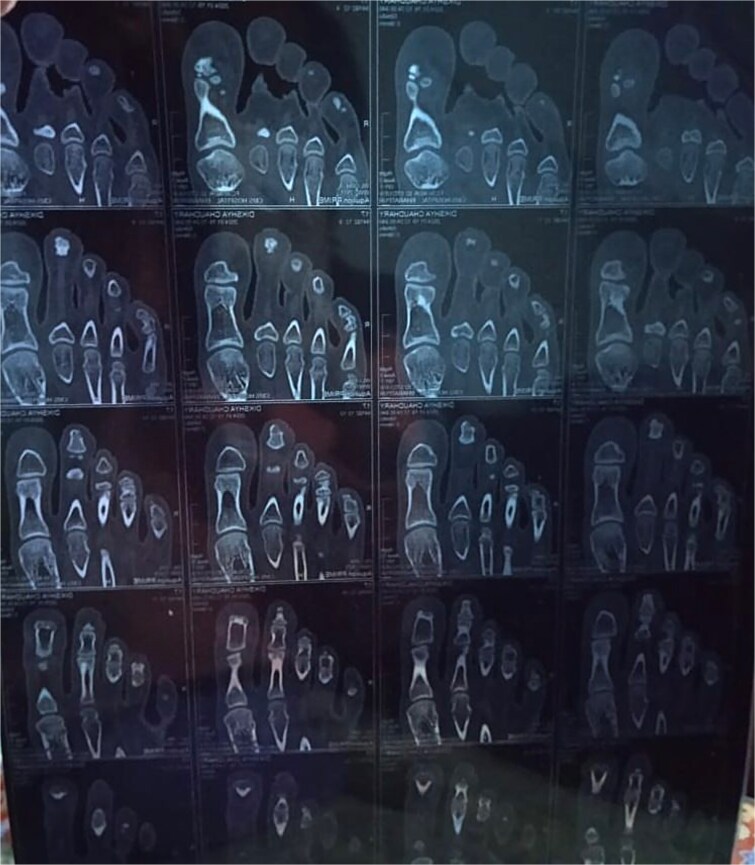
CT scan of right foot revealing a linear undisplaced fracture over distal phalanx of second toe.

Routine blood investigations revealed normal findings. Plain X-ray of right foot appeared normal. CT scan of the right foot showed a linear undisplaced fracture of the distal phalanx of the second toe with minimal adjacent soft tissue swelling (Fig. 2). The patient was referred to the orthopaedic department, where she was advised to rest the foot, and splinting of the affected toe was done.

## Discussion

Digital clubbing, first described by Hippocrates, is also known as Hippocratic fingers/toes, drumstick fingers/toes, or watch-glass nails [1]. The clubbed digits are mostly asymptomatic; however, they often predict the presence of some serious underlying diseases [1]. Clubbing is usually bilateral and acquired, seen in cardiac, pulmonary, and gastrointestinal disorders, but may occur in congenital or familial forms [1, 3]. Congenital nail clubbing is mostly symmetrical and bilateral; however, varying degrees of involvement of fingers and toes may occur. Familial form of clubbing, with autosomal dominant or recessive form of inheritance, can be due to mutation in HPGD or SLCO2A1 gene [4, 5].

Unilateral clubbing is usually associated with impaired regional blood flow caused by localized vascular lesions of the arm, axilla, and thoracic outlet, recurrent shoulder dislocation, or Pancoast tumor [1, 3]. Single-digit clubbing is a rare condition usually caused by expansive lesions or injury in the distal phalanx [2, 3]. The causes of single-digit clubbing reported in the literature are myxoid cyst, osteoid osteoma, enchondromas, myxochondromas, superficial acral fibromyxoma, sarcoidosis, median, and ulnar nerve injury [2, 6]. The fracture leading to hypoxia and release of platelet-derived growth factor (PDGF) and vascular endothelial growth factor (VEGF) might be responsible for inducing clubbing in our patient [1, 3]. We suggest an occult fracture should be considered in the differential diagnosis of clubbing in a single digit. The proper radiographic studies should be carried out in single-digit clubbing to rule out an underlying disorder.

Unidigital clubbing is extremely rare, so this case is being reported to enlighten the clinician with an uncommon clinical sign, which will aid in early diagnosis and prompt management of the underlying condition.

## Data Availability

The data that support the findings of this study are available from the corresponding author upon reasonable request.
